# Nanoparticles prepared from porous silicon nanowires for bio-imaging and sonodynamic therapy

**DOI:** 10.1186/1556-276X-9-463

**Published:** 2014-09-03

**Authors:** Liubov A Osminkina, Vladimir A Sivakov, Grigory A Mysov, Veronika A Georgobiani, Ulyana А Natashina, Florian Talkenberg, Valery V Solovyev, Andrew A Kudryavtsev, Victor Yu Timoshenko

**Affiliations:** 1 Department of Physics, Lomonosov Moscow State University, 119991, Moscow, Russia; 2 Leibniz Institute of Photonic Technology, Jena 07745, Germany; 3 Institute of Theoretical and Experimental Biophysics, RAS, Pushino 142290, Russia

**Keywords:** Silicon nanowires, Silicon nanoparticles, Photoluminescence, Bio-imaging, Cytotoxicity, Sonodynamic therapy, Theranostic

## Abstract

Evaluation of cytotoxicity, photoluminescence, bio-imaging, and sonosensitizing properties of silicon nanoparticles (SiNPs) prepared by ultrasound grinding of porous silicon nanowires (SiNWs) have been investigated. SiNWs were formed by metal (silver)-assisted wet chemical etching of heavily boron-doped (100)-oriented single crystalline silicon wafers. The prepared SiNWs and aqueous suspensions of SiNPs exhibit efficient room temperature photoluminescence (PL) in the spectral region of 600 to 1,000 nm that is explained by the radiative recombination of excitons confined in small silicon nanocrystals, from which SiNWs and SiNPs consist of. On the one hand, *in vitro* studies have demonstrated low cytotoxicity of SiNPs and possibilities of their bio-imaging applications. On the other hand, it has been found that SiNPs can act as efficient sensitizers of ultrasound-induced suppression of the viability of Hep-2 cancer cells.

## Background

Recently, a lot of bio-applications of different silicon nanostructures were reported. The most popular material in this field of interest is porous silicon (PSi). PSi consists of a network of intersecting silicon nanocrystals (nc-Si) separated by nanometer-sized pores [1]. Usually, PSi films are formed by a method of electrochemical etching of bulk crystalline silicon (c-Si) in hydrofluoric acid (HF), which was firstly showed in 1956 by Uhlir [2]. The size of nc-Si and pores in PSi depends on the formation parameters, such as HF concentration, current density, and substrate doping density [1,3]. In [4-6], it has been shown that PSi, which consists of nc-Si with sizes of 2 to 5 nm, demonstrates efficient photoluminescence (PL) in the visible spectral range at room temperatures, originating from quantum confinement in nc-Si [7]. In 1995, Canham [8] discussed bio-friendly properties of PSi and suggested its applications in bio-medicine. Such properties of PSi and Si-based nanoparticles (SiNPs) as bio-compatibility and bio-degradability have been previously investigated [9,10]. SiNPs can be used as luminescent labels [10-12], nanocontainer for drug delivery [13,14], sensitizers of photo- [15-18] and ultrasound irradiation (USI) therapy [19,20]. Furthermore, other bio-medical applications of SiNPs were also proposed [21-26].

It is known a cheap and efficient method of the formation of PSi and silicon nanowires (SiNWs) based on metal-assisted chemical etching (MACE) of c-Si [27-31]. MACE-prepared SiNWs consists of an almost non-intersecting nanowires with diameters from several to hundreds nm [28]. The morphology of the as-synthesized SiNWs was highly dependent on the doping level of original silicon wafers and on the concentrations of etching solutions [32]. SiNWs show unique physical properties such as room temperature PL [33,34], enhanced Raman scattering [35], low reflectance in the visible spectral range, and a strong broadband optical absorption [31,36] and gained much attention due to their possible applications in the fields of photovoltaics, photocatalysis, gas sensors, lithium-ion battery, and drug delivery carriers [33,37]. Also, a long-term antiseptic effect of SiNWs decorated with silver nanoparticles was shown in [38].

In this paper, we propose a simple method for the preparation of SiNP suspensions, which is based on grinding of SiNWs in water by ultrasound irradiation. The prepared SiNPs are used as PL probes for bio-imaging and sensitizers for sonodynamic suppression of the cancer cells proliferation *in vitro*.

## Methods

SiNWs were prepared by MACE of heavily boron-doped (doping level 10^20^ cm^-3^; conductivity < 0.005 Ω cm) (100) single crystalline wafers. Prior to the MACE procedure, the Si substrates were rinsed in 5% HF aqua solution for 1 min to remove the native oxide. Then, in the first step of MACE, thin (approximately 100 nm) layers of Ag nanoparticles of different morphology were deposited on the substrates by immersing them in aqueous solution of 0.02 M of silver nitrate (AgNO_3_) and 5 M of HF in the volume ratio of 1:1 for 30 s. In the second step, the Si substrates covered with Ag nanoparticles were immersed in the solution containing 5 M of HF and 30% H_2_O_2_ in the volume ratio of 10:1 in a teflon vessel for 20 min. The etching was performed at room temperature. Then, SiNW arrays were rinsed several times in deionized water and additionally immersed in concentrated (65%) nitric acid (HNO_3_) for 15 min to remove the residual Ag nanoparticles from the SiNWs. Finally, the samples were rinsed several times in deionized water and dried at room temperature. Aqueous suspensions of SiNPs were prepared by 3 h ultrasound grinding (37 kHz, 90 W) of SiNWs. Afterward, the suspensions were centrifuged for 3 min at 2,000 rpm, and the resulting supernatant was used in the experiments. The *in vitro* experiments were done with freshly prepared SiNP suspensions to avoid an influence of the dissolution of SiNPs in water.

Structural investigations of SiNW-based samples were carried out by using a field emission scanning electron microscope (Carl Zeiss ULTRA 55 FE-SEM, Oberkochen, Germany) and a transmission electron microscope (LEO 912 AB OMEGA, Oberkochen, Germany). A Malvern Zetasizer Nano ZS (Malvern Instruments Ltd., UK) instrument was used to determine the size and zeta potential (ZP) of SiNPs from the dynamic light scattering (DLS) data. The PL was excited by the radiation of an Ar^+^ -ion laser at 364 nm (power 10 mW, spot diameter 1 mm). The PL signal was detected using a grating monochromator (MS750, SOLAR TII, Moscow, Russia) equipped with a CCD array. The external quantum yield (QY) of PL was measured by using a calibrated detector as it was described in [39]. *In vitro* cytotoxicity experiments were performed with Hep-2 human lung cancer cells. The cells were incubated with SiNPs and cultural medium (Dulbecco’s modified Eagle medium, Biolot, St. Petersburg, Russia) for 24 h. The cancer cells were directly counted in a hemocytometer by using the standard method when dead cells were separated from living ones by preliminary coloring with trypan blue (0.4%, 1:1) (PanEco Ltd., Moscow, Russia). The results were statistically processed.

*In vitro* SiNPs visualization experiments were carried out with CF2Th (dog thymus) cells infected with a green fluorescent protein (GFP) gene. Thirty hours before the fluorescence analysis, RSL-1 inducer was added to the CF2Th culture, which caused the synthesis of GFP. The luminescence wavelength of GFP is near 515 nm (green light). SiNPs were introduced into CF2Th 5 h after the inducer injection and 24 h before the measurements. Cell nuclei were imbued with 5 μg Hoechst (PanEco Ltd., Moscow, Russia) 30 min before the experiment. The luminescence wavelength of Hoechst is near 460 nm (blue light). The cells with incorporated SiNPs were studied using a Leica TCS SP5 confocal microscope (Wetzlar, Germany).

To evaluate the effect of ultrasound and SiNPs on the Hep-2 cell viability, SiNPs in the cultural medium were added to the cells to achieve the nanoparticles concentration 0.1 mg/ml and left for 14 h prior to the experiment. Afterward, the cells were washed three times by Hanks’ balanced salt solution (PanEco Ltd., Moscow, Russia) and removed from the substrate by trypsinization. The obtained suspension of cells was exposed to USI (0.88 MHz, 1 W/cm^2^, pulse mode, modulation 2/20), with the UST-1.3.01 F ‘MeDTeKo’ equipment. Degassed distilled water (at 37°C) was used as a contact medium between flat emitters with a radius of 2 cm and cuvette filled with the sample. In the control group, the cells without SiNPs were investigated.

## Results and discussion

**Figure 1:**
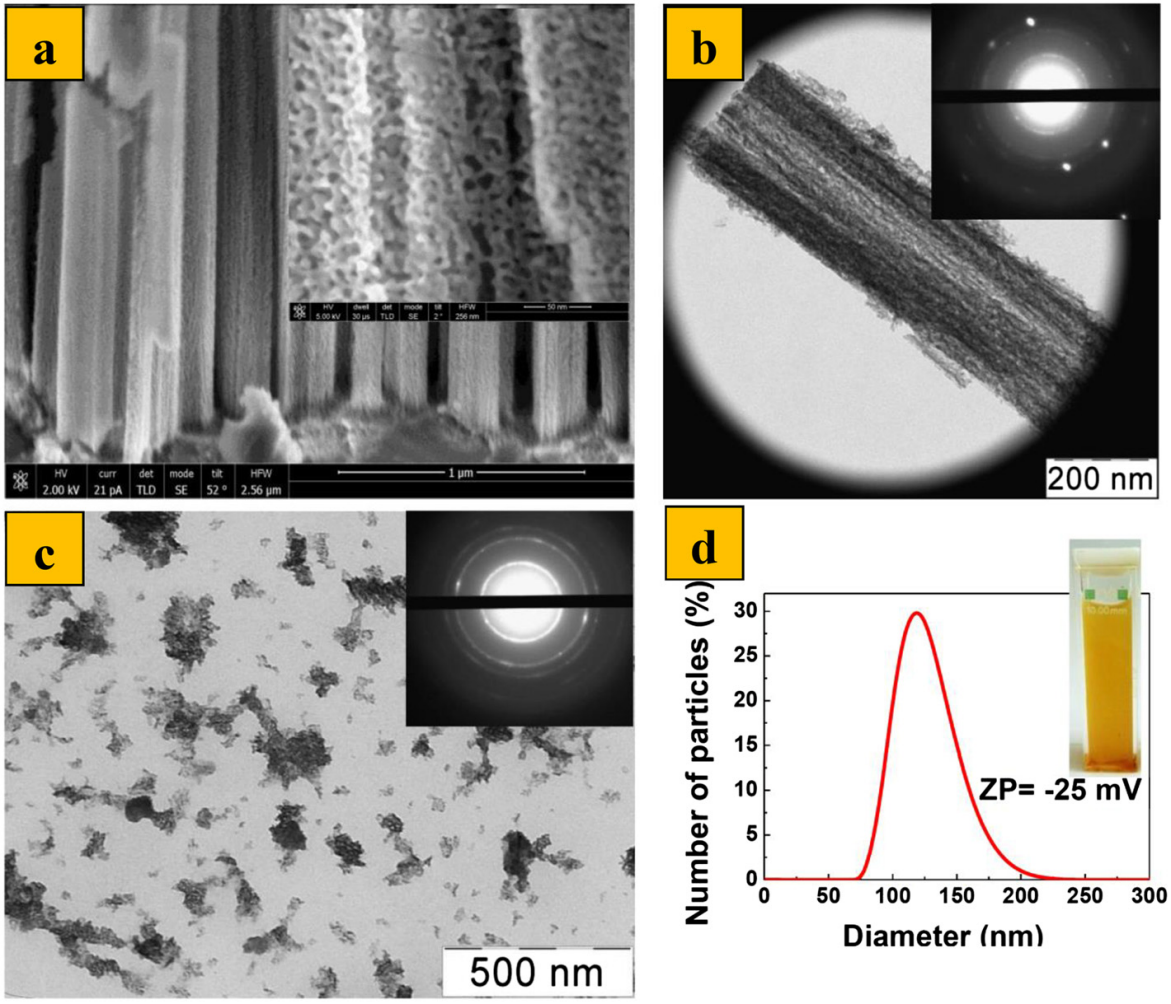
**SiNWs and SiNPs structural characterization. (a)** Cross-sectional SEM image of SiNW arrays on c-Si (the inset shows the porous structure of the SiNW), **(b)** TEM image of single SiNW (the inset shows the electron diffraction pattern of the SiNW), **(c)** TEM image of SiNPs (the inset shows the electron diffraction pattern of the SiNPs), and **(d)** size distribution function of SiNPs, obtained by DLS (the inset shows the digital photo of the SiNPs aqueous suspension with the concentration of 1 mg/ml).

**Figure 2:**
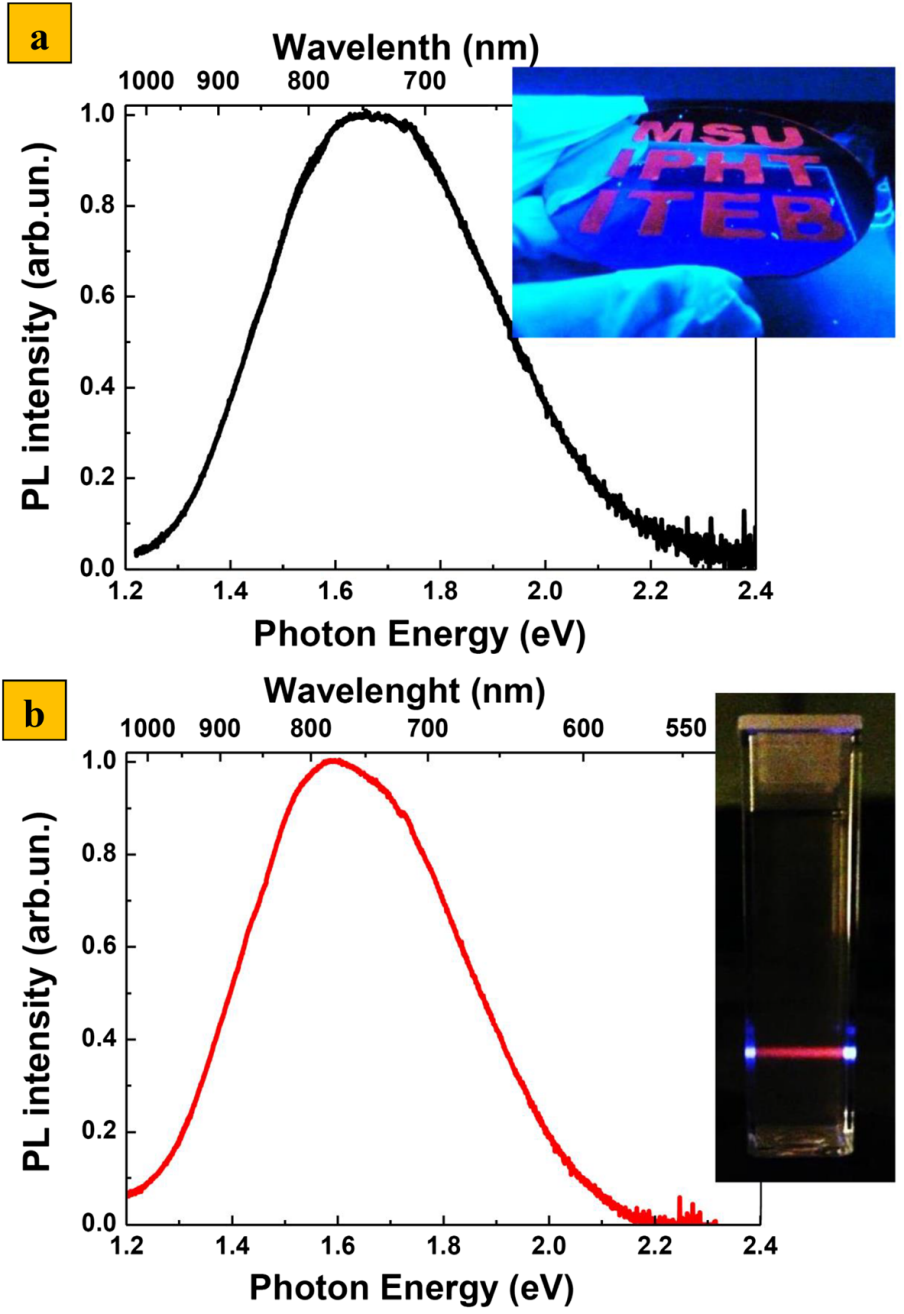
**Photoluminescence properties of SiNWs and SiNPs. (a)** PL spectra of SiNW layers (inset: digital photo of silicon wafer structured by SiNWs with participating institution letters under UV excitation) and **(b)** PL spectra of aqueous suspensions of SiNPs prepared from SiNWs (inset: typical appearance of the SiNP suspension under the UV line of argon-laser radiation).

Figure 1a shows typical cross-sectional scanning electron microscopy (SEM) micrographs of heavily doped SiNW arrays on c-Si. Inset in Figure 1a shows a porous structure of SiNW. Quasi-ordered similarities in SiNW arrays with preferential orientation along the (100) crystallographic direction have been observed. The length of SiNWs was observed to be about 5 μm after 20 min etching time. As you can see from SEM (inset in Figure 1a), SiNWs have a highly porous structure, which is also confirmed by the transmission electron microscopy (TEM) image of single SiNW as presented in Figure 1b. Such porous microstructure of nanowires is typical for SiNWs obtained from MACE etching of highly doped c-Si [32]. The diameter of SiNW is about 200 nm, and it is nearly constant through the whole SiNW length.Inset in Figure 2b shows the corresponding electron diffraction pattern obtained in the ‘transmission’ geometry for SiNWs. The presence of rings in the pattern indicates the preservation of misoriented nanocrystals in the porous structure of SiNWs.

A TEM image of SiNPs, fabricated by ultrasound grinding of SiNWs in water, is presented in Figure 1c. Inset shows the electron diffraction pattern for SiNPs, which indicates the preservation of misoriented nanocrystals in SiNPs. Figure 1d shows a size distribution function of SiNPs, obtained by DLS, which was characterized by a maximum of 135 nm. ZP of SiNPs in the initially obtained aqueous suspensions was -20 ± 2 mV. The ZP value is typical for porous silicon nanoparticles and related to the negative charge of hydroxyl groups on SiNPs surfaces [40]. Such negative ZP determines the existence of stable suspensions of nanoparticles. A typical view of SiNP aqueous suspension with the concentration of 1 mg/ml is presented in the inset of Figure 1d. Note that the Raman spectra (not shown) of as-prepared SiNWs and dried SiNP suspensions showed that both samples consisted of Si nanocrystals with mean size of 3 to 4 nm. A negligible fraction of the amorphous silicon was also found. The preservation of nanocrystalline structure of the samples after the preparation of SiNW suspension can be explained by the effect of surface oxide formed during the last stage of MACE and nitric acid treatment.

The PL spectra of SiNWs consist of a broad band in the visible range with the maximum in the photon energy at 850 nm (1.7 eV) as shown in Figure 2a. The external QY of PL was estimated to be about 2%, and the PL emission could be easily observed with a naked eye (see the inset in Figure 2a). The PL spectra of the samples are well explained by the radiative recombination of excitons confined in small silicon nanocrystals with an average size of 2 to 6 nm [31]. Such nanocrystals were found by electron microscopy in porous volume of SiNWs as shown in Figure 1a,b.The intensity and character of the PL spectra of SiNPs suspensions is similar to the corresponding SiNW layers, and their PL could be easily observed with a naked eye (see the inset in Figure 2b). As mentioned above, the 2 to 6 nm nanocrystals are responsible for this PL. Thus, it can be argued that the obtained SiNPs have a porous structure Therefore, the SiNWs milling procedure does not significantly quenching their PL properties, and obtained PL SiNPs can be used in different bio-medical application, in particular, for cells labeling.

**Figure 3:**
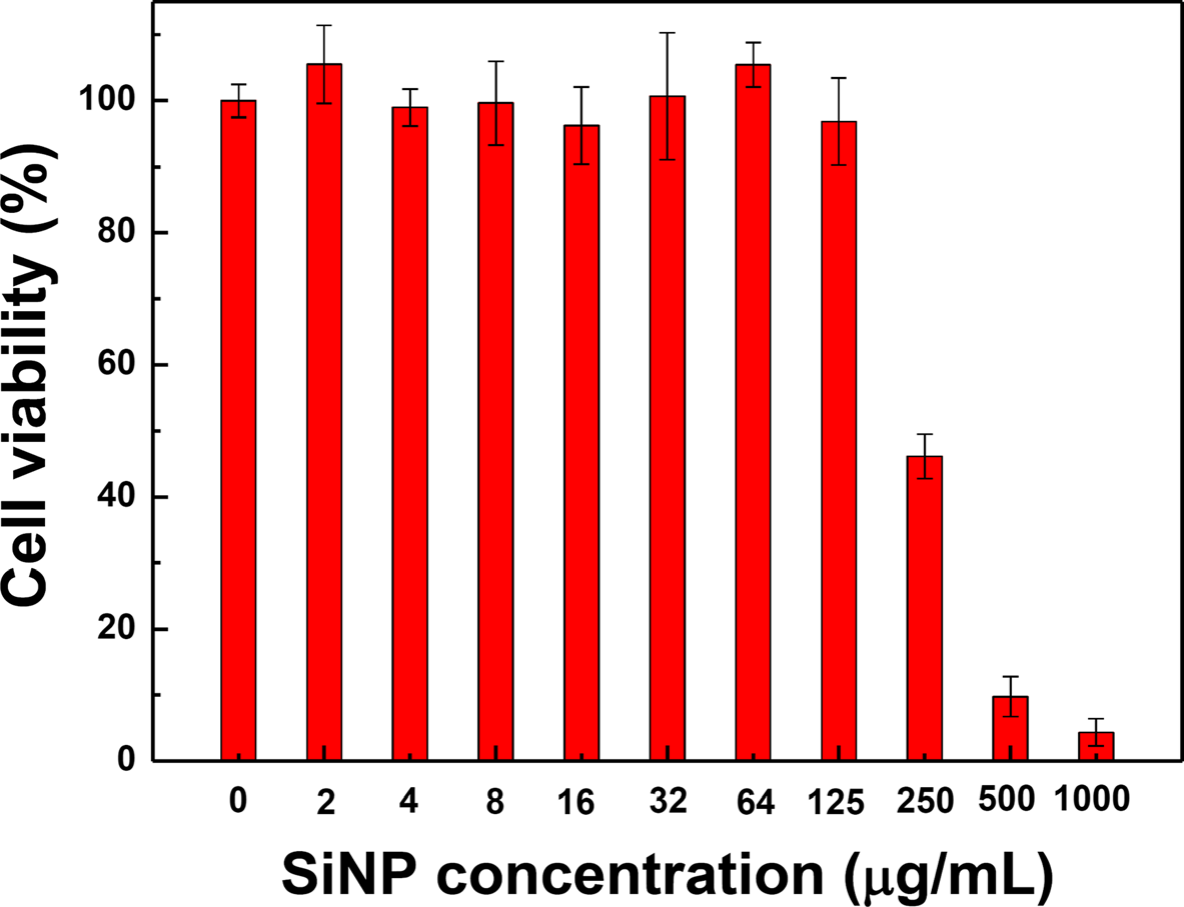
***In vitro *** **cytotoxicity of SiNPs towards living Hep-2 cells.**

Figure 3 shows *in vitro* cytotoxicity of SiNPs towards living Hep-2 cells. As it is clearly visible, SiNPs are relatively non-toxic to Hep-2 cells in the concentration range of 2 to 125 μg/ml. However, at larger concentrations of SiNPs, of 250 to 1,000 μg/ml, the cell viability was dropped to 5% as shown in Figure 3.

**Figure 4:**
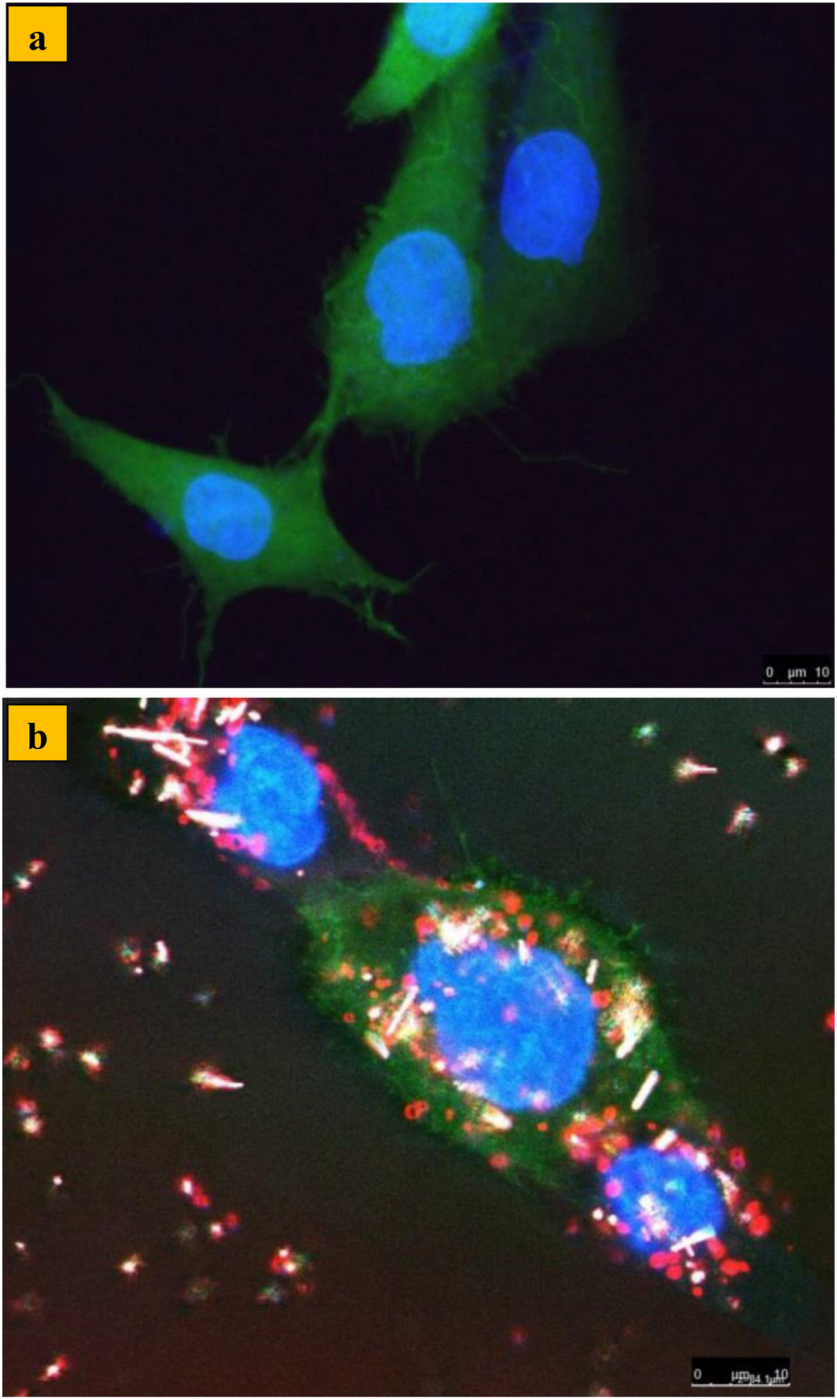
***In vitro *fluorescence images of living CF2Th cells. (a)** In the control group and **(b)** with SiNPs. Green, blue, and red colors correspond to the luminescence of cell membranes, cell nuclei and SiNPs, respectively. The images scale bar is 10 μm.

The *in vitro* fluorescence photographs of living CF2Th cells with and without (the control) introduced 0.1 mg/ml SiNPs are presented in Figure 4a,b, respectively. Green, blue, and red colors correspond to the luminescence of cell membranes, cell nuclei and SiNPs, respectively.

A significant luminescence of SiNPs was observed in the cells 24 h after their incubation. It is evident that the majority of SiNPs could penetrate into the cells and locate in their cytoplasm. We assume that nanoparticles penetrate into the cells due to the mechanism of endocytosis [41]. The localization of SiNPs in the cell cytoplasm was confirmed by z-scan imaging [see Additional file 1: Movie S1].

**Figure 5:**
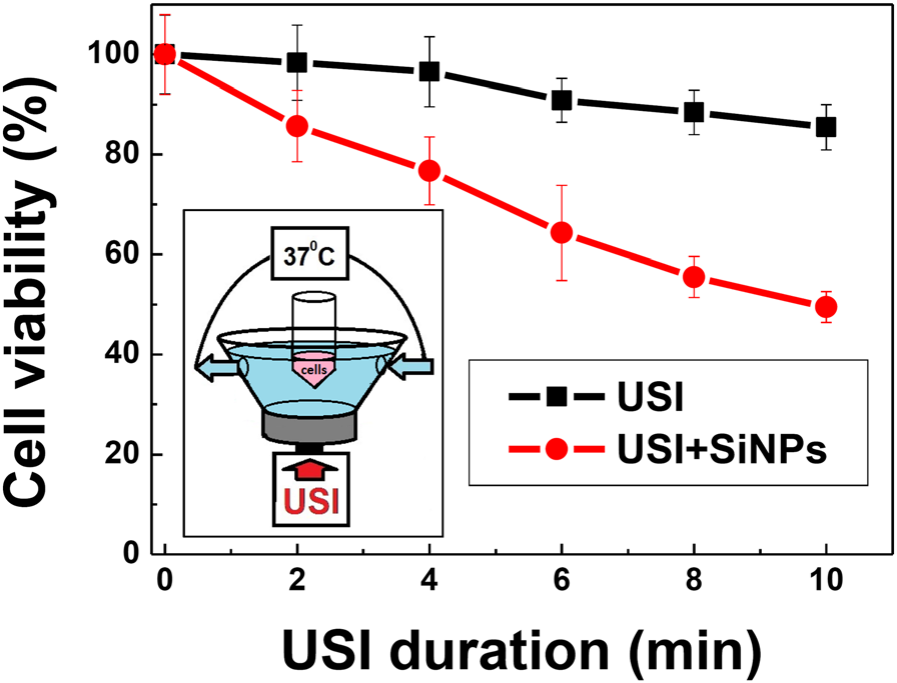
**The dependence of *in vitro *cell viability on USI duration.** The cells have been exposed to USI (black curve) or to the combined action of USI and SiNPs (red curve). Inset: schematic representation of the setup for *in vitro* USI experiments.

Figure 5 represents the living cell viability dependence on USI duration. Setup of *in vitro* experiments is given in the inset of Figure 5. It was found out that USI essentially no affect on the cell viability within the time interval from 0 to 10 min. At the same time, the combined action of ultrasound and SiNPs led to 50% drop in the number of living cells as compared to the control.

The effect of ultrasound and SiNPs on the cell viability can be explained by the appearance of local increasing of temperature near SiNPs, so-called hyperthermia, caused by the adsorption of the ultrasound energy [42]. Also, the possibility of appearance of cavitation near the nanoparticles under ultrasound irradiation cannot be excluded.

The obtained results open a new perspective for the application of bio-compatible SiNPs, produced from ultrasound milling of porous SiNWs, in SDT.

In summary, low toxicity, photoluminescence and sonosensitizing properties of SiNPs, prepared by ultrasound milling of porous SiNW arrays, open new possibility of their theranostic (therapy and diagnostic) applications.

## Conclusions

We have shown that aqueous suspensions of SiNPs can be obtained by a simple and cheap way, i.e., by ultrasound milling of porous SiNW arrays in water. After 2 h of grinding, SiNPs represent a mixture of 200 nm porous agglomerates composed of 2 to 6 nm nc-Si. The prepared PL SiNPs are characterized by low cytotoxicity *in vitro* even at concentrations of 125 μg/ml and can be used for bio-imaging of cancer cells. The prepared SiNPs also exhibit the properties of efficient sonosensitizer of therapeutic ultrasound. It was found out that the combined action of ultrasound and SiNPs with concentration of 100 μg/mL led to 50% decrease in the number of cancer cells. The obtained results open a new perspective for the usage of bio-compatible porous SiNPs prepared from SiNWs in the sonodynamic therapy of cancer.

## Competing interests

The authors declare that they have no competing interests.

## Authors’ contributions

LAO performed the optical measurements and data analysis. VAS contributed in the development of the preparation method and analysis of the results. GAM and VAG performed the measurements of the PL spectra of the samples. UAN performed the structural measurements and the DLS spectra of the samples. FT performed the SiNWs fabrication. VVS and AAK performed *in vitro* measurements. VYT performed the general data analysis and discussion of the obtained data. All authors participated in writing the manuscript and approved its final version.

## Supplementary Material

Additional file 1 **(‘Z-scan movie of living CF2Th cells with added SiNPs’) shows a z-scan movie of living CF2Th cells with added SiNPs.** Green, blue, and red colors correspond to the luminescence of cell cytoplasm (colored with GFP), cell nuclei (colored with Hoechst) and SiNPs, respectively. Click here for file
